# Undernotification and underreporting of tuberculosis in Zambia: a national data quality assessment

**DOI:** 10.1186/s12913-022-08431-2

**Published:** 2022-08-22

**Authors:** P. S. Lungu, M. E. Kabaso, R. Mihova, A. Silumesii, T. Chisenga, C. Kasapo, I. Mwaba, A. D. Kerkhoff, M. Muyoyeta, R. Chimzizi, K. Malama

**Affiliations:** 1 Ministry of Health, Lusaka, Zambia; 2 USAID Sustaining Technical and Analytic Resources (STAR) Project, Lusaka, Zambia; 3 USAID Eradicate TB Program, Lusaka, Zambia; 4 Centre for Infectious Disease Research in Zambia, Lusaka, Zambia; 5 Division of HIV, Infectious Diseases and Global Medicine, Zuckerberg San Francisco General Hospital and Trauma Center University of California, San Francisco, San Francisco, CA USA

**Keywords:** Tuberculosis, Notification, Zambia, Quality improvement, Underreporting

## Abstract

**Background:**

Despite national implementation of several high impact interventions and innovations to bolster tuberculosis (TB) detection and improve quality of TB services in Zambia, notifications have been declining since 2004. A countrywide data quality assessment (DQA) of Zambia’s National TB and Leprosy Programme (NTLP) was undertaken to quantify the degree to which undernotification and underreporting of TB notifications may be occurring.

**Methods:**

The NTLP conducted a retrospective DQA of health facilities in high burden districts in all ten Zambian provinces. Multiple routine programmatic data sources were triangulated through a multi-step verification process to enumerate the total number of unique TB patients diagnosed between 1st January and 31st August 2019; both bacteriologically confirmed and clinically diagnosed TB patients were included. Undernotification was defined as the number of TB patients identified through the DQA that were not documented in facility treatment registers, while underreporting was defined as the number of notified TB cases not reported to the NTLP.

**Results:**

Overall, 265 health facilities across 55 districts were assessed from which 28,402 TB patients were identified; 94.5% of TB patients were ≥ 15 years old, 65.1% were male, 52.0% were HIV-positive, and 89.6% were a new/relapse case. Among all TB cases, 32.8% (95%CI: 32.2–33.3) were unnotified. Undernotification was associated with age ≥ 15 years old (adjusted prevalence odds ratio [aPOR] = 2.4 [95%CI: 2.0–2.9]), HIV-positive status (aPOR = 1.6 [95%CI: 1.5–1.8]), being a new/relapse TB case (aPOR = 17.5 [95%CI: 13.4–22.8]), being a clinically diagnosed TB case (aPOR = 4.2 [95%CI:3.8–4.6]), and being diagnosed at a hospital (range, aPOR = 1.5 [95%CI: 1.3–1.6] to 2.6 [95%CI: 2.3–2.9]). There was substantial heterogeneity in the proportion of unnotified TB cases by province (range, 18.2% to 43.6%). In a sub-analysis among 22,199 TB patients with further data available, 55.9% (95%CI: 55.2–56.6) were notified and reported to the NTLP, 32.8% (95%CI: 32.2–33.4) were unnotified, and 11.3% (95%CI: 10.9–11.7) went unreported to the NTLP.

**Conclusions:**

The findings from Zambia’s first countrywide TB programme DQA demonstrate substantial undernotification and underreporting of TB cases across all provinces. This underscores the urgent need to implement a robust and integrated data management system to facilitate timely registration and reporting of all TB patients who are diagnosed and treated.

## Introduction

Globally, an estimated 4 million cases of tuberculosis (TB) are missed each year due to missed diagnoses and undernotification/underreporting of TB patients [1]. Missed diagnoses may reflect persons with TB either not seeking care or being unable to access care, or a failure by health systems to recognise them as presumptive TB cases [2–4]. Limited access to highly sensitive diagnostic tools at lower levels of care where persons with TB often first present for evaluation of their symptoms may also contribute to missed diagnoses [5, 6]. On the other hand, undernotification occurs when patients that are diagnosed with TB are not recorded in the official facility treatment register, while underreporting reflects TB patients initiated on TB treatment and recorded in the facility register, but not accounted for in reports submitted to the national level [7]. Missed diagnoses and undernotification/underreporting both create challenges for estimating the true burden of TB and also controlling its spread [8–11]. Therefore, it is important to determine factors contributing to missed TB cases in order to better target interventions and allocate resources [12, 13].

With an estimated TB incidence of 333 cases per 100,000 population, Zambia is among the top 30 high burden TB countries globally [1]. Since 2004, the number of notified TB cases has been declining [1, 14]. However, a national TB prevalence survey that was conducted between 2013 and 2014 revealed that the burden of TB was higher than previously estimated and that the decline in notification was likely due to low case detection [15]. Using the results of the prevalence survey, WHO subsequently revised upwards the incident TB estimates for Zambia. In 2019, Zambia had an estimated TB burden of 59,000 cases, of which only 61% were estimated to have been diagnosed and started on treatment [1].

To better quantify the extent to which low TB case detection and treatment coverage may be due to missed diagnoses or undernotification and underreporting, the Zambian National TB and leprosy program (NTLP) undertook a national level inventory study, known as a “data quality assessment, (DQA).” Inventory studies are recommended by the World Health Organisation (WHO) to measure the degree of TB undernotification and underreporting in TB programmes [7]. The DQA was conducted at health facilities in each of Zambia’s 10 provinces to better understand factors contributing to declining TB notifications, which has led to the NTLP not reaching its annual targets since 2015 – the year in which WHO TB incidence estimates for Zambia were revised upward based upon the results of the most recent national TB prevalence survey. The DQA also aimed to assess how TB data was managed at each level (facility, district, province, and national level) and how each of these levels report TB data to higher levels (e.g., data reporting structures). In this manuscript we report the findings from a countrywide DQA of Zambia’s national TB programme.

### Methodology

A retrospective data quality assessment was conducted by the NTLP under routine programme conditions between September and November 2019. This was the first countrywide data quality assessment exercise to be conducted in Zambia since the reorganization of the TB programme in 2000. The assessment covered all TB patients diagnosed between 1^st^ January and 31^st^ August 2019; this included adults and children, those who had bacteriologically confirmed or clinically diagnosed TB, and new, relapse, and retreatment TB patients.

### Health records and TB notification system in Zambia

The National TB program of Zambia uses a paper-based system for recording; all cases are recorded within one of three registers. All patients being investigated for TB are captured in the presumptive TB register and the laboratory register. Once a diagnosis of TB is made, a patient is notified, and a patient is only deemed to have been notified if their record has been captured in the TB treatment register and card. The TB treatment register and card are the sources for aggregate notification data that is sent from the facility to the district, through to the province and finally to the national level. Any patient who is not captured in the treatment register or card will neither be reported in the national notification system, nor subsequently to WHO. In addition to the registers described above, all TB patients attending health facilities have their care captured in clinical records including doctors’ notes, ward rounding books, and antenatal clinic forms and registers; these shall henceforth be referred to as facility health records. As is the case with TB registers, facility health records in Zambia are almost entirely paper-based.

### Selection of Districts and Health facilities

Zambia has ten provinces, and each province is divided into administrative districts. Diagnostic and treatment centres from all ten provinces of Zambia were included in the DQA. Sixty priority districts out of 117 total districts in Zambia were purposively selected by the DQA technical team. The selection criteria included: 1) historical high TB notifications; 2) high HIV prevalence; 3) high population density; 4) presence of mining activities and large correctional facilities and 5) being a border district. Collectively, the selected districts accounted for 94% of TB notifications in 2018.

**Fig. 1:**
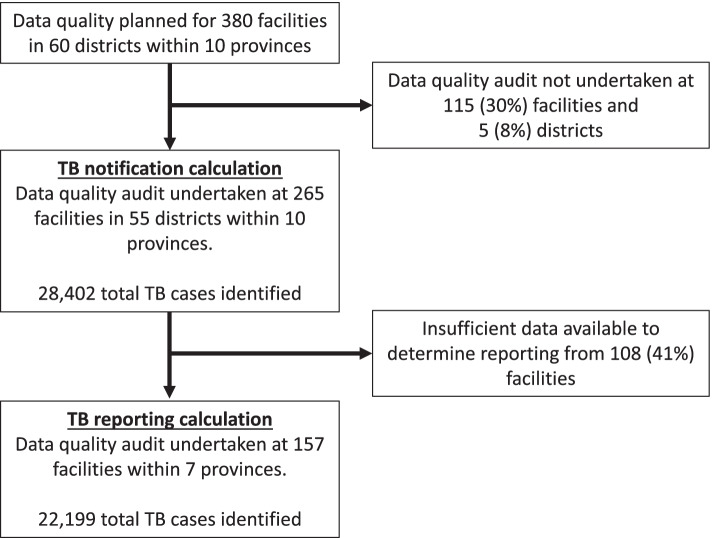
Flow diagram of the national TB programme data quality assessment (DQA) in Zambia

Within each of the 60 selected priority districts, all primary healthcare facilities that also served as TB diagnostic centres, as well as level 1 (smaller hospital with few clinical specialties; District level) 2 (mid-size hospital with some clinical specialties; Provincial level) and 3 (large hospital that is highly specialized; National level) hospitals were included. Additional private facilities in the Lusaka and Copperbelt Provinces were also included. In total, 380 health facilities from 60 districts were selected for the DQA (Fig. 1).

### Data collection procedures

We used three electronic excel sheets designed to collect data on all patients diagnosed with TB at 1) outpatient department and patients admitted in the wards, 2) in TB treatment registers and cards kept at the TB clinic and 3) in TB laboratory registers. TB patients recorded in the TB treatment registers kept at the TB clinic were entered in an excel sheet named “LIST A”. TB patients documented in registers, forms and books kept at outpatient departments (OPD) and inpatient departments (IPD) were captured in an excel sheet named “LIST B.” The records of all TB patients in the TB laboratory register were abstracted into a sheet coded as "LIST C”. All data collection tools were pretested before use in the field. At each participating health facility, data was abstracted from paper-based health facility records by an experienced NTLP staff member and entered directly into electronic excel data collection tools as described above.

### Data aggregation

Several steps were then undertaken to aggregate and analyse the data. To assess undernotification at each facility, the audit team compiled data on all TB patients found at the OPD, IPD and TB laboratory and compared it with the data on TB patients recorded in the facility TB treatment registers. First, the total number of notified patients for each facility was quantified from the official treatment registers and cards using list A (TB treatment register). Next, all patients with a recorded diagnosis of TB from OPD and IPD were quantified using list B. Patients with a recorded TB detected result in the laboratory register were quantified using list C. Patients appearing on both clinically diagnosed and bacteriologically confirmed cases as well as those documented twice in the same register were de-duplicated before data analysis to avoid double counting and under-estimation of the proportion of unnotified data.

To assess under- or overreporting, routinely reported notification data was compared to verified notification data. Reported data were notifications that were routinely reported through facility- and district-level NTLP Excel reports and District Health Information System 2 (DHIS2), while verified data were the TB notifications that were verified as part of the DQA. Comparisons between routinely reported and DQA-verified notifications were undertaken at every level (facility, district and provincial); if differences were found this was noted as a gap (over- or under-) and if no difference was found, this was reported as “passed validation.”

### Definitions and analysis

“TB diagnosis” was defined as having a ‘TB detected result’ recorded in the laboratory (bacteriologically confirmed) register or having a diagnosis of TB recorded in the patient records (clinically diagnosed). “New TB” cases were defined as patients with no known prior diagnosis of or treatment for TB; “relapse TB cases” were defined as patients who previously had a prior TB episode and were classified as completed treatment or cured and whom are diagnosed with a new TB episode; “retreatment TB cases” were defined as patients who were treated for a prior TB episode, but either failed treatment, were lost-to-follow up, or had an unknown outcome, and represent for treatment of the same TB episode. “Notification” was defined as having a diagnosed TB case registered in the official facility-specific TB notification and treatment register. “Undernotification” was defined as the total number of patients found in the facility health records (including laboratory registers) with a documented TB diagnosis that were not recorded in the official treatment register at the facility (e.g., list B + C – list A). “Underreporting” was defined as the total number of TB cases found in the official facility notification treatment registers that were not reported to the to the national level. Therefore, while all notified TB cases are either classified as reported or unreported, by definition, all TB cases that are unnotified are also unreported.

Simple descriptive statistics were used to characterize all identified TB cases. The proportion of undernotification was determined by dividing the total number of unnotified cases (the numerator) by the total number of TB patients found in all facility health records (e.g., notified plus unnotifed cases; the denominator). The proportion of underreporting was determined by dividing the total number of unreported TB cases (the numerator) by the total number of TB patients found in all facility health records (e.g., notified plus unnotifed cases; the denominator). Unadjusted prevalence odds ratios (POR) were calculated to determine characteristics and factors associated with under notification of TB cases. These results informed a multivariable model where all covariates in the univariable model meeting a predetermined *P*-value cutoff of ≤ 0.1 were included. All analyses were undertaken using SPSS Statistics (Version 21, IBM Corp., Armonk, NY, USA) and Stata (Version 17, Stata Corp., College Station, TX, USA).

## Results

### Districts and facilities audited

**Fig. 2:**
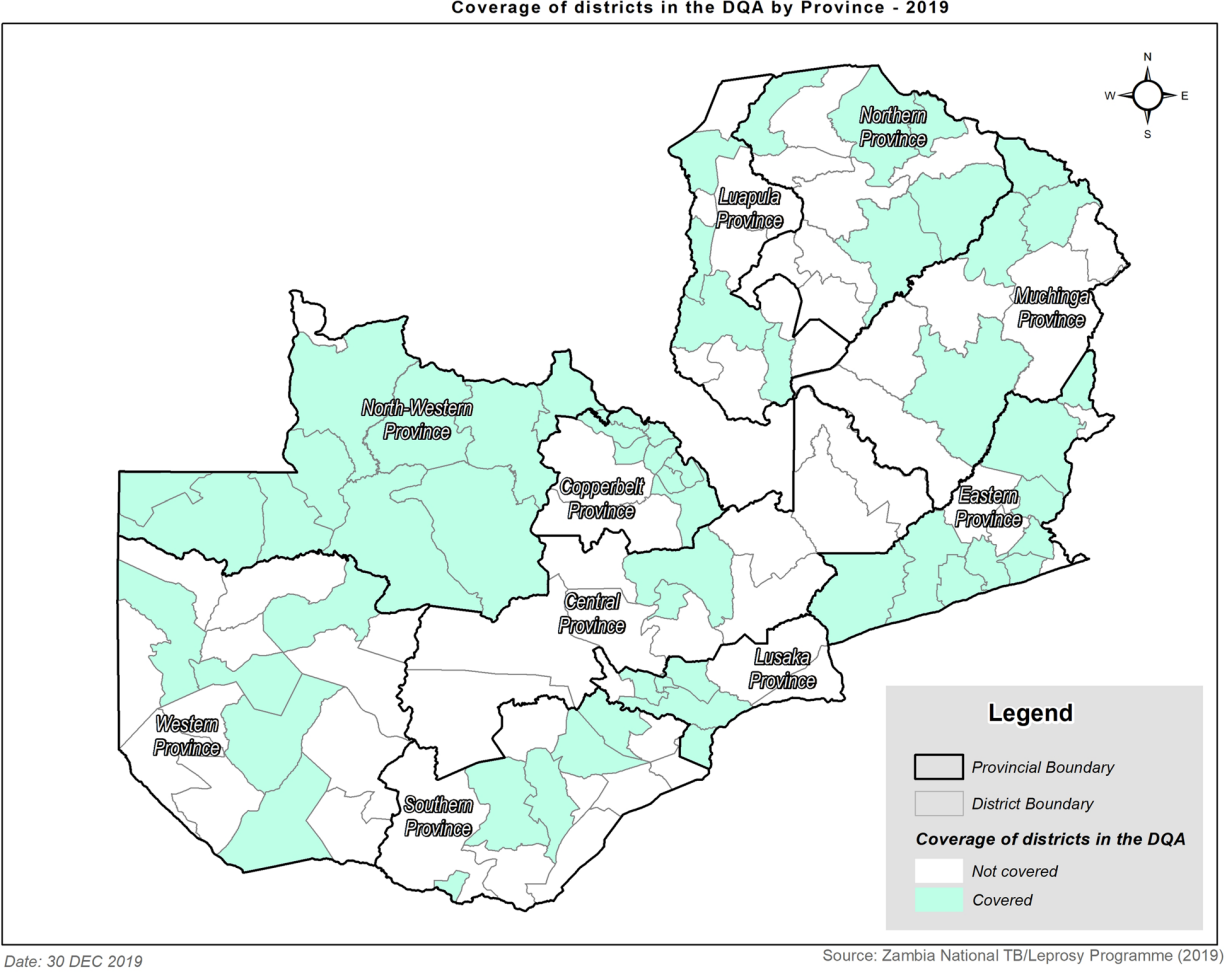
Geospatial map showing the location of facilities assessed by district during the national TB data quality assessment (DQA)

Out of the 380 planned health facilities in 60 districts distributed across the ten provinces of Zambia, 265 health facilities in 55 districts were audited, representing a coverage of 69.7% and 91.6% for the planned health facilities and districts, respectively (Table 1, and Fig. 1). Not all planned districts and facilities could be audited due to time constraints and long physical distances between facilities that would have substantially increased costs. The geographic distribution of the facilities that were covered during the audit is shown in Fig. 2. Out of the 10 provinces in Zambia, 52.5% of audited facilities were in Copperbelt or Lusaka provinces.

**Table 1:** Planned and actual districts and facilities audited by province

Province	Planned Coverage			Actual Coverage		Proportion of planned coverage (%)	
	**Districts**	**Facilities**	**Districts**		**Facilities**	**Districts**	**Facilities**
**Central**	8	26	2		19	25%	73%
**Copperbelt**	9	138	8		87	89%	63%
**Eastern**	5	33	7		10	140%	30%
**Luapula**	5	17	4		12	80%	71%
**Lusaka**	5	45	5		52	100%	116%
**Muchinga**	4	11	4		9	100%	82%
**Northern**	6	17	5		13	83%	76%
**North-Western**	5	16	10		25	200%	156%
**Southern**	8	39	5		19	63%	49%
**Western**	6	38	5		19	83%	50%
**Overall**	**60**	**380**	**55**		**265**	**92%**	**70%**

### Total TB cases identified during DQA

A total of 28,402 TB patients were identified through the DQA of which 94.5% were adolescents/adults aged 15 years or older, 65.1% were male, 52.0% were HIV-positive and 3.9% died (Table 2). The large majority (89.6%) of TB patients represented a new or relapse case; 52.7% of all TB cases were bacteriologically confirmed, while 47.3% patients were clinically diagnosed. Nearly half (48.6%) of TB cases were diagnosed at primary healthcare facilities, a quarter (24.9%) were diagnosed at level 1 hospitals, while the remaining were diagnosed at level 2 and 3 hospitals (Table 2).

**Table 2:** Characteristics of tuberculosis patients identified during data quality audit of 265 health facilities in Zambia according to notification status

Patient Characteristics		Total tuberculosis cases identified		Notified tuberculosis case		Unnotified tuberculosis case		Unadjusted prevalence odds ratio for unnotified TB case (95%CI)	*P*-value	Adjusted prevalence odds ratio for unnotified TB case (95%CI)	*P*- value
		***n***	***%***	***N***	***%***	***n***	***%***				
**All Cases**		**28,402**		**19,094**		**9,308**					
**Age group** **(in years)**	0—14	1,556	5.5	1,197	6.3	359	3.9	1.0		1.0	
	≥ 15	26,846	94.5	17,897	93.7	8,949	96.1	1.67 (1.48–1.88)	< 0.001	2.38 (1.97–2.88)	< 0.001
**Sex**	Female	9,922	34.9	6,593	34.5	3,329	35.8	1.0		1.0	
	Male	18,480	65.1	12,501	65.5	5,979	64.2	0.95 (0.90–1.00)	0.040	1.09 (1.01–1.19)	0.033
**HIV Status**	Negative	9,914	48.0	9,094	49.6	820	35.2	1.0		1.0	
	Positive	10,738	52.0	9,229	50.4	1,509	64.8	1.81 (1.66–1.98)	< 0.001	1.60 (1.45–1.76)	< 0.001
	Unknown	7,750	27.3	771	4.0	6,979	75.0	100.39 (90.56–111.30)	< 0.001	147.97 (131.11–166.99)	< 0.001
**Patient type**	Retreatment case	2,952	10.4	2,848	14.9	104	1.1	1.0		1.0	
	New/relapse cases	25,450	89.6	16,246	85.1	9,204	98.9	15.51 (12.74–18.90)	< 0.001	17.48 (13.38–22.83)	< 0.001
**Method of diagnosis**	Bacteriologically confirmed	14,967	52.7	10,848	56.8	4,119	44.3	1.0		1.0	
	Clinically diagnosed	13,435	47.3	8,246	43.2	5,189	55.7	1.66 (1.58–1.74)	< 0.001	4.21 (3.82–4.64)	< 0.001
**Vital Status**	Alive	27,300	96.1	18,527	97.0	8,773	94.3	1.0		1.0	
	Died	1,102	3.9	567	3.0	535	5.7	1.99 (1.77–2.25)	< 0.001	2.22 (1.86–2.64)	< 0.001
**Facility Level**	Primary healthcare facility	13,796	48.6	10,331	54.1	3,465	37.2	1.0		1.0	
	Level 1 Hospital	7,077	24.9	4,931	25.8	2,146	23.1	1.30 (1.22–1.38)	< 0.001	1.48 (1.34–1.64)	< 0.001
	Level 2 Hospital	3,253	11.5	2,083	10.9	1,170	12.6	1.67 (1.54–1.82)	< 0.001	1.62 (1.43–1.84)	< 0.001
	Level 3 Hospital	4,276	15.1	1,749	9.2	2,527	27.1	4.31 (4.01–4.63)	< 0.001	2.57 (2.30–2.89)	< 0.001

### TB notifications and characteristics associated with unnotified cases

**Fig. 3:**
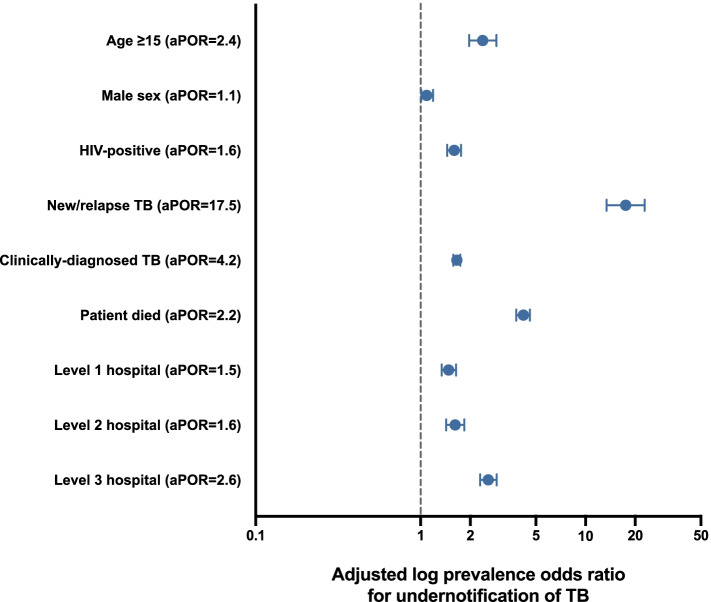
Forest plot showing independent risk factors associated with TB patients identified during the national TB data quality assessment (DQA) being unnotified in a local facility TB notification and treatment register. aPOR = adjusted prevalence odds ratio

**Fig. 4:**
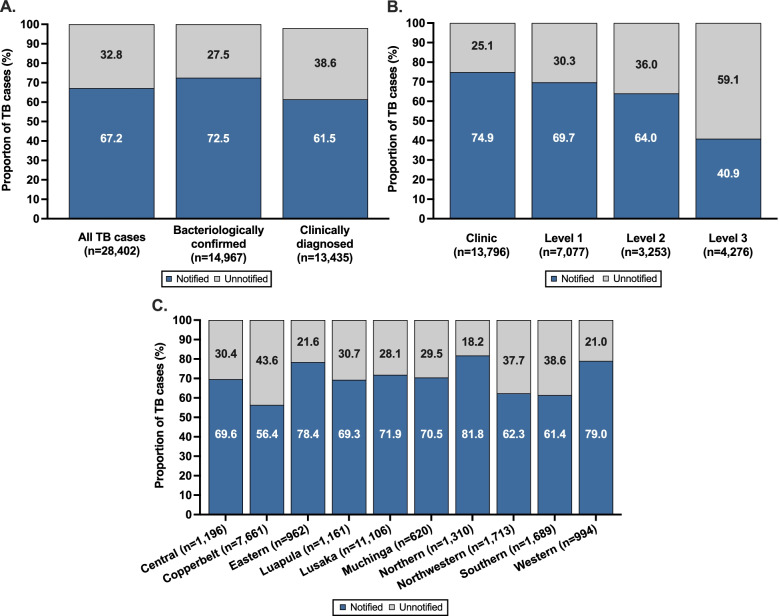
The proportion of total TB patients identified during the national TB data quality assessment (DQA) that were recorded in TB notification and treatment registers (notified) according to: **a** TB case type, **b** facility type and **c** province

Overall, 9,308 (32.8% [95%CI: 32.2–33.3] of the 28,402 TB cases identified during the DQA were unnotified. TB patients aged ≥ 15 years old (adjusted POR [aPOR]) = 2.4 [95%CI: 2.0–2.9]), had a higher odds of being unnotified than children with TB. HIV-positive TB persons (aPOR = 1.6 [95%CI: 1.5–1.8]) and persons with an unknown HIV status (aPOR = 148.0 [95%CI: 131.1–167.0]) had a higher odds of being unnotified compared to HIV-negative TB persons (Fig. 3). Men with TB (aPOR = 1.1 [95%CI: 1.0–1.2]) had a slightly higher odds of being unnotified than women with TB (Table 2). TB patients who died prior to hospital discharge had a higher odds than those who remained alive to be unnotified (aPOR = 2.2 [95%CI:1.9–2.6]). New or relapsed TB patients had a very high odds of being unnotified (aPOR = 17.5 [95%CI: 13.4–22.8]) when compared to retreatment TB patients. While clinically diagnosed TB comprised only 47.3% of all identified TB cases, they accounted for 55.7% all unnotified cases (Table 2) and were substantially more likely to be unnotified (38.6% [95%: 37.8–39.5] vs. 27.5% [95%CI: 26.8–28.2]; aPOR = 4.2 [95%CI:3.8–4.6]) compared to bacteriologically confirmed TB cases (Fig. 3 and 4a).

### TB undernotification by facility type and province

Despite level 3 hospitals contributing to only 15.1% of all identified TB patients, they accounted for 27.1% of all unnotifed TB patients. Notably, 59.1% of TB patients diagnosed at a level 3 hospital were unnotified, which was a substantially higher proportion than TB patients diagnosed at lower-level health facilities (Fig. 4b, Table 2); compared to TB patients identified at primary healthcare facilities, TB patients at level 3 hospitals had 4.3-times higher odds of being unnotified (Fig. 3).

Large differences in the proportion of TB cases that were unnotified were observed by province and ranged from 18.2% to 43.6% (Fig. 4c). In Copperbelt Province, which accounted for the second most TB cases in Zambia (27.0% of all identified cases), 43.6% of TB cases were unnotified, while in Lusaka Province, which accounted for the most TB cases in Zambia (39.1% of all identified cases), 28.1% of TB cases were unnotified. Collectively, these two provinces accounted for 6,457 (69.4%) of all unnotified TB patients.

### Reporting of notified TB cases to the national TB programme

**Fig. 5:**
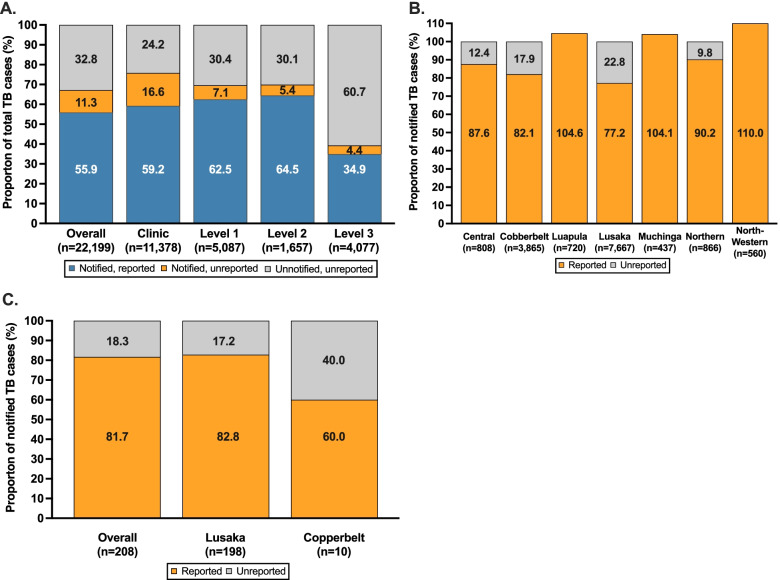
Assessment of underreporting of notified TB cases in Zambia. Panel **A** shows the proportion of all TB cases identified during the national TB data quality assessment (DQA) that were notified and reported, notified but unreported, or were unnotifed (and unreported). Panel **B** shows the proportion of all notified TB patients that were verified during the national TB data quality assessment (DQA) that were reported to the National TB Programme at public health facilities according to province. Panel **C** shows the proportion of all notified TB patients that were verified during the national TB data quality assessment (DQA) that were reported to the National TB Programme at private health facilities according to province

Next, we sought to evaluate the degree to which notified TB cases may be underreported to the NTLP; there were 157 facilities across 7 provinces with sufficient data available at the time of the DQA to determine the proportion of notified TB cases that were reported (Fig. 1). Overall, 22,199 TB patients were identified, of which 55.9% (n = 12,409, 95%CI: 55.2–56.6) were notified (and documented in facility-level TB treatment registers), and were also reported to the national level (Fig. 5a); diagnosed TB patients who were unnotified (and thus were also not reported to the national level) accounted for 32.8% (*n* = 7,276, 95%CI: 32.2–33.4) of all cases, whilst 11.3% (*n* = 2,514, 95%CI: 10.9–11.7) of TB cases were notified but not reported to the NTLP. Among the 14,923 notified TB cases identified, 82.5% (*n* = 12,409, 95%CI: 83.2–83.8) were reported, while 16.8% (*n* = 2,514, 95%CI: 16.2–17.5) were not reported. The proportion of notified, but unreported TB cases was highest at primary healthcare facilities (16.6%) and lowest at level 3 hospitals (4.4%) (Fig. 5a).

### Underreporting and overreporting by province within the public and private sector

Substantial heterogeneity was observed by Province with respect to reporting of notified TB cases to the NTLP. Copperbelt (17.9%) and Lusaka (22.6%) provinces were found to have large proportions of unreported TB cases while Luapula, Muchinga and Northwestern provinces all reported a larger number of cases to the national reporting systems than were notified and recorded in the TB treatment facility registers (Fig. 5b).

Of the 10 private hospitals that were audited (9 in Lusaka Province and 1 in Copperbelt Province), 208 notified TB patients were found, of which 81.7% (*n* = 170, 95%CI: 76.0- 86.7) were reported to the NTLP while 18.3% (*n* = 38, 95%CI: 13.3–24.2) were unreported (Fig. 5c).

## Discussion

The findings from Zambia’s first national TB programme DQA demonstrate that there is substantial undernotification and underreporting of TB cases across Zambia’s 10 provinces. Countrywide, the level of undernotifications of TB patients at health facilities and underreporting of notified TB cases to the national level was found to be 33% and 11%, respectively; therefore, only 56% of all TB cases identified in this DQA were notified and reported. The magnitude of underreporting identified in the DQA was also comparable to the findings of other inventory studies conducted in the region from Malawi, South Africa and Kenya [8, 16, 17].

Undernotification varied substantially by facility type and was most prevalent at level 3, tertiary referral hospitals where 59% of all identified TB patients were unnotified, compared to 25% of those attending lower level, local primary healthcare facilities. In Zambia, TB treatment services are offered in a designated area of the health facility (referred to as the TB corner or chest clinic), which is typically physically separated from other departments and there is minimal integration of services. Currently, TB patients diagnosed and started on anti-TB drugs while hospitalized inpatients, especially at level 3 hospitals, are only notified at the time of discharge from the hospital. Upon discharge, TB patients are expected to pass through the chest clinic for notification. This may contribute to undernotification of TB cases in several ways: 1) TB patients who start treatment, but die before being discharged are never notified; 2) some TB patients who are discharged from the hospital over the weekend or outside of working hours may not be notified because chest clinics only operate between 8 am and 5 pm and are closed over the weekends; 3) due to incomplete knowledge or a misunderstanding, some TB patients may leave the hospital and present to local lower level health facilities without first stopping at the hospital’s chest clinic to be notified; 4) the chest clinic may be far from hospital wards and/or difficult to find, which serves as a possible physical barrier to TB notification. While such TB patients are commonly entered into their local primary healthcare facility’s treatment register, they are not reported to the NTLP so as to avoid duplicate case reporting; thus, they are not accounted for at all levels of care.

To address these weaknesses, TB diagnosis and treatment services need to be coordinated and fully integrated across departments and facilities with clear communication and reporting structures between more centralized, higher-level hospitals and local community primary healthcare facilities. One possible strategy would be for higher level facilities (e.g., level 3 hospitals), which make the primary TB diagnosis and initiate TB treatment, to assume responsibility for ensuring that each patient is notified and that notification data is verified before transmitting reports to the district level; standardized trainings and refresher trainings on data management and reporting procedures and structures are likely to be a key component of such an approach. Further, one important systems-based strategy would be to de-implement the current paper-based reporting structure while simultaneously implementing an electronic reporting structure, which includes a unique patient identifier for each person who is diagnosed with and started on TB treatment. This would facilitate reduced data entry errors, improve data entry efficiency and would allow the NTLP to undertake ongoing, real-time data-quality assessment at all levels of care.

Clinically diagnosed TB patients were more likely to be unnotified (39%), but it was surprising that 28% of bacteriologically confirmed TB diagnoses went unnotified. This observation provides some evidence that microbiological-confirmation of TB diagnoses, in part through continued scale-up of and improved access to rapid, highly sensitive TB diagnostic tools (e.g., Xpert MTB/RIF or Ultra) may help to improve linkage to care and reduce undernotification. Unfortunately, the DQA was not able to systematically determine what proportion of unnotified patients (clinically diagnosed or bacteriologically confirmed) were never started on anti-TB therapy (e.g., pre-treatment lost-to-follow-up) and what proportion were started on ant-TB therapy but were not documented in the facility treatment register. Insight gained while undertaking the DQA suggests that while the majority of unnotified TB patients are likely being linked to care and provided life-saving treatment, there also likely remains an important minority of persons with TB who are lost-to-follow-up before initiating anti-TB therapy.

Pre-treatment lost-to follow-up is common in high burden TB settings and has previously been demonstrated to range between 4 to 38% among TB patients [18]. This may reflect a number of factors such as fear, anxiety and stigma associated with TB following a new diagnosis, missing or incorrect contact information to call patients back to the facility to initiate treatment, long TB test turnaround times and patients that may have subsequently moved away, a reliance in some settings for the patient themselves to facilitate their own linkage to treatment services, and the direct and indirect costs that may make returning to the healthcare facility to start TB therapy difficult for some patients [18–20]. The DQA results also demonstrated that death prior to initiation of anti-TB therapy remains an important cause of pre-treatment lost-to-follow-up in this setting—49% of TB patients identified and known to have died (most of whom were noted to have died within 48 h of diagnosis) were not notified compared to 32% who were alive at the time the DQA was undertaken. Further, in Zambia, due to multiple competing priorities, busy laboratories do not actively inform the requesting clinician of positive TB results and the busy clinicians often do not actively follow up results of the patient’s sputum they ordered. In addition to strengthening data reporting capacity and structures, the DQA results suggest a need to identify effective and sustainable strategies that may reduce pre-treatment lost-to-follow-up and in-turn, reduce the number of unnotified TB patients.

In addition to substantial undernotification of TB patients, the DQA also found that that 17% of notified TB patients and 11% of all identified TB cases were not reported to the NTLP (underreporting). This provides further evidence that number of persons with TB who are accessing treatment in Zambia is substantially higher than prior recent national and international reports [1]. Similar to findings from Kenya [8], the highest levels of underreporting were observed in predominantly urban settings (Lusaka and Copperbelt provinces). While the private health sector in Zambia is thought to account for a very small proportion of TB diagnoses and treatment initiations countrywide, the DQA identified that the private sector has high levels of underreporting (18% of notified cases), mirroring trends observed in the public health sector. Factors identified that contributed to underreporting included: transposition errors – for example, one facility reported 15 rather 51 notifications, some facilities did not report any data for an entire quarter—resulting in substantial under-estimates, and other facilities submitted identical reports across quarters—resulting in under-estimates as more TB cases were identified in the subsequent quarter. While most provinces were found to be underreporting TB cases, it was an important finding that some provinces (predominantly rural) were also overreporting TB cases, albeit somewhat small in magnitude (e.g., 5–10%). Nonetheless, the heterogenous findings of both under- and overreporting across settings, sectors and provinces, further suggests the need to improve the management of TB data, including the complete integration of private facilities into national TB reporting systems.

There were several strengths associated with this study. First, the DQA included more than 250 health facilities, both public and private, at all levels, across 55 districts and all 10 provinces – this represented nearly 90% and 70% of planned districts and facilities. However, the DQA did not evaluate facilities in districts with a low number of notifications and/or not representing at least one key epidemiological risk factor for TB in Zambia and thus the results of the DQA may not be generalizable to such facilities. Nonetheless, the DQA included facilities that accounted for nearly 95% of all TB notifications in the prior year and therefore we believe that the DQA findings representative of TB data quality in Zambia. Also, pre-tested data collection tools were utilized to standardize data collection procedures across diverse settings. There were however some limitations. While DQA coverage was high, we were unable to reach all planned facilities and districts due to logistical considerations including inadequate time and long distances between facilities that would have increased costs. Additionally, the DQA utilized routine programmatic data, which was at times incomplete or could not be found, especially individual patient files and death records. Further, Zambia shares national borders with eight countries, and we were unable to determine the contributions of cross-border migration on undernotification and underreporting. Finally, we were able to assess notification but not reporting levels for three provinces due to differences in how the data was aggregated during reporting across levels.

In conclusion, the first countrywide DQA of Zambia’s TB programme revealed substantial undernotification and underreporting across all provinces and suggest higher levels of TB detection and treatment coverage than previously estimated. These findings underscore the urgent need to implement a robust and integrated data management system to facilitate timely registration and reporting of all persons with TB who are diagnosed and initiated on treatment.

## Data Availability

The data are not publicly available; however, the government of Zambia allows data sharing when applicable local conditions are satisfied. To request data access please contact the corresponding author.
